# Effects of Using Child Personas in the Development of a Digital Peer Support Service for Childhood Cancer Survivors

**DOI:** 10.2196/jmir.7175

**Published:** 2017-05-18

**Authors:** Pontus Wärnestål, Petra Svedberg, Susanne Lindberg, Jens M Nygren

**Affiliations:** ^1^ School of Information Technology Halmstad University Halmstad Sweden; ^2^ School of Health and Welfare Halmstad University Halmstad Sweden

**Keywords:** peer, childhood, cancer, survivor, participation, user experience, service design

## Abstract

**Background:**

Peer support services have the potential to support children who survive cancer by handling the physical, mental, and social challenges associated with survival and return to everyday life. Involving the children themselves in the design process allows for adapting services to authentic user behaviors and goals. As there are several challenges that put critical requirements on a user-centered design process, we developed a design method based on personas adapted to the particular needs of children that promotes health and handles a sensitive design context.

**Objective:**

The purpose of this study was to evaluate the effects of using child personas in the development of a digital peer support service for childhood cancer survivors.

**Methods:**

The user group’s needs and behaviors were characterized based on cohort data and literature, focus group interviews with childhood cancer survivors (n=15, 8-12 years), stakeholder interviews with health care professionals and parents (n=13), user interviews, and observations. Data were interpreted and explained together with childhood cancer survivors (n=5) in three explorative design workshops and a validation workshop with children (n=7).

**Results:**

We present findings and insights on how to codesign child personas in the context of developing digital peer support services with childhood cancer survivors. The work resulted in three primary personas that model the behaviors, attitudes, and goals of three user archetypes tailored for developing health-promoting services in this particular use context. Additionally, we also report on the effects of using these personas in the design of a digital peer support service called Give Me a Break.

**Conclusions:**

By applying our progressive steps of data collection and analysis, we arrive at authentic child-personas that were successfully used to design and develop health-promoting services for children in vulnerable life stages. The child-personas serve as effective collaboration and communication aids for both internal and external purposes.

## Introduction

### Peer Support for Childhood Cancer Survivors

Advances in diagnosis, risk stratification, and treatment protocols have resulted in that most children who are diagnosed with cancer survive the disease and have the potential to live a long life with a quality of life comparable with their peers. However, the transition from a period of intensive treatment to everyday life is often associated with physical and psychological problems for which they need professional support [1-3]. Managing these physical and psychological problems as well as the social challenges that are associated with the experiences and consequences of the disease can be facilitated by social support mediated by peers on whom the child can rely and who share a similar background [4]. The availability of such peer support is limited and resources are often offered sporadically and by the initiative of patient organizations or health care services [5]. This shortage in resources that fulfills the needs and requirements of the target group demonstrates the need to develop peer support services that are adapted to the preferences and requirements of child users and that are not restricted by clinical, geographical, age-related, and logistic limitations of face-to-face interaction associated with this user group [6].

### Designing With and for Children in a Sensitive Context

Developing digital peer support (DPS) services directed toward children surviving from cancer to facilitate health-promoting social connectedness to other children with similar experiences has to involve the children themselves in the design process to allow for inclusion of their perspectives on challenges of survival and on how to integrate such services into their everyday life [7,8]. A user-centered design (UCD) process enables designers to meet the children’s worldview, their cognitive and emotional developmental stage, age, and gender [9], as well as their requirements on usability and experiential quality [6]. Children can be involved through a variety of UCD techniques and methods such as cooperative and participatory design [10,11], contextual design [12], and persona-based design [13-15]. The involvement of children can be achieved through participation in different stages of the design process and for different purposes such as, defining the needs within the user group in a particular area, formulating the aim of the design, planning and setting up methodologies, assembling and analyzing data, sketching and prototyping, and planning and implementing dissemination of findings [16].

Involvement of children who have survived cancer in the context of health-promoting and social digital services is associated with several challenges, which, if unaddressed, might have a negative impact on their chances of getting an active role in a user-centered design process. First, although it is acknowledged that children have the right to be heard in matters that affect their lives, health, and care, they are often viewed as “vulnerable” subjects due to their dependence on adults. It is therefore essential to protect children from harm associated with involvement and therefore carefully handle consent to participation, confidentiality, research context, and activities [17]. Second, children who have experiences from a severe disease are seen as even more vulnerable in relation to participation and it is common that various gatekeepers restrict their participation, such as ethical boards, health care professionals, parents [18], and ultimately, the children themselves and their willingness to participate in processes developed by researchers or designers [17]. Third, the specific characteristics of this user group (eg, age-span, medical history, geographic spread, and clinical restrictions) make it difficult to recruit, meet, and engage the children on a regular basis for long-term participatory design work. UCD techniques and methods that take these considerations into account could be powerful tools for user involvement in the design context outlined previously. To address these problems, we developed a design process based on “personas” adapted to the particular requirements of addressing the needs of children, promoting health, and handling a sensitive context.

### A Persona-Based Design Process for Health Care Contexts

Persona-based design is a UCD approach that provides a vivid representation of the target users, makes them concrete and life-like, and not merely described by demographic statistics. Personas are defined as “fictitious, specific, concrete representations of target users” [19]; however even though they are fictitious, personas are created directly from research data collected using both qualitative and quantitative research methods. The use of personas as a design method has been applied in different ways. One of the most well-known in the industry is goal-directed design (GDD) [13,14]. GDD has been used within health care research projects as well [20].

An essential benefit of using personas is that they have the potential to build empathy for the target users [21]. In the context of designing interactive systems, empathy is understood as an understanding of—and identification with—the user to ensure that they will be able to take advantage of the service being designed and will be able to use it with pleasure rather than frustration. Another benefit often voiced regarding persona-based design is the persona’s effectiveness in conveying design requirements to various stakeholders [22,23]. However, as we predicted that several of the stakeholders would act as gatekeepers and—based on a protective stance—would limit access to children with experience from cancer treatment in the development of a DPS service, we applied an approach toward users’ participation based on differences between a “salutogenic” orientation and a “pathogenic” orientation in relation to health. The original idea of salutogenesis states that it is more important to focus on peoples’ resources and capacities to create health rather than focusing on risks, ill health, and disease (pathogenesis) [24]. The concept of salutogenesis has been developed toward, first, problem solving and second, capacity to use resources available [25]. In line with this we used the salutogenic orientation to focus the development of a DPS service on factors that maintain and promote health and well-being of childhood cancer survivors and the pathogenic orientation to focus on factors that are related to disease and treatment in relation to DPS services. The salutogenic orientation was thus used to incorporate aspects that are inherent to the users and their motivations, behaviors, and goals related to everyday life at home, with friends and family, and in school, whereas the pathogenic orientation was used to incorporate stakeholders’ views on the challenges and limitations related to disease and treatment that a DPS service has to deal with.

### Study Aim

Along these lines, our design approach takes a user-centered, cocreative perspective, where the use of child-personas plays an important role. The design context for children who have survived cancer is of a sensitive nature, where ethical considerations need to be addressed regarding what topics can be handled during interviews and workshops to ensure a healthy environment for all participants [26]. This puts additional requirements on the design methodology, as the restrictions inherent in this sensitive design context limit the ways user data can be collected compared with traditional approaches. The purpose of this study was to develop high-quality, authentic personas for DPS service design for children who have survived cancer. This paper describes and discusses a method for coconstruction of personas with children in sensitive design contexts and the effects of using these personas in the development of a DPS service for childhood cancer survivors.

## Methods

### Study Design

Due to ethical reasons, a salutogenic perspective was prominent throughout the activities performed with children. Stakeholder interviews with health care professionals and parents of children with a history of cancer provided a pathogenic perspective. Personas were later created to merge these perspectives. The study was approved by the regional ethical board (dnr 2011/307).

### Participants

For the initial semistructured focus group interviews, children between the ages of 8-12 that were currently under maintenance treatment or in remission from cancer were recruited (Table 1). Children that were still in an acute state of cancer treatment and children who had only received radiation therapy or surgery were excluded from the study. The children were recruited through two Swedish hospitals. Nurses selected 10 girls and 19 boys that fit the inclusion criteria, of whom 5 girls and 10 boys consented to participate. Both the children and their parents gave informed consent.

Recruitment for the stakeholder interviews was based on an organizational perspective with the identification of important professional roles (eg, medical specialist, clinic leader, consultancy nurses; Table 1). From the initial interviews, additional important roles were identified. In total, 9 health care professionals were interviewed: 1 medical specialist or doctor, 1 clinic leader (doctor), 1 oncology nurse, 2 consultancy nurses, 2 play or occupational therapists, 1 sibling supporter (nurse), and 1 representative from a local childhood cancer patient association. Additionally, 4 parents of children between the ages of 9-11 years with a history of cancer were interviewed.

From the 15 children who had participated in the initial focus group interviews, 5 were chosen for further participation in a series of 3 explorative design workshops (Table 1). These children (3 boys aged 11-12 years and 2 girls aged 11 and 13 years) were selected as they represented particularly well-functioning groups and had shown a high level of creativity during the initial focus group interviews. For the final workshop, 3 girls and 4 boys without experience from cancer treatment aged 10-12 years were recruited from a local school (Table 1).

### Focus Group Interviews

The focus group interviews aimed at researching how children with a history of cancer treatment experienced friendship [27] (Table 1). The focus of the interviews was how the children interacted socially with their friends and what their everyday lives looked like. The focus groups (n=5) each comprised 3 children of similar age and same gender and were performed in a location chosen together with the children’s families. In this context, small homogeneous groups are recommended to ensure that children’s needs are met [18,28]. Due to geographical reasons, one of the focus group interviews was performed with groups of children with mixed genders.

The focus group interviews, each approximately 90 min, were recorded using video and sound. The interviews were semistructured and separated into three stages. First, a section meant to create familiarity within the group and familiarize the children with each other [29]. Second, a section where the children used creative and informative techniques to articulate opinions and experiences on relevant topics. Activities included, for example, brainstorming, drawing and telling, listing, and answering activity-oriented questions—techniques used to help children in expressing themselves [30,31]. Finally, a third closing section that summarized the focus group interview.

### Stakeholder Interviews

Considering input from subject-matter experts and important business roles is a vital and common precursor to successful design. These interviews are often performed before user involvement. Also, for this study we were aware of the limitation of not being able to broach the pathogenic perspective with the children, and thus further importance was placed on these interviews. The semistructured stakeholder interviews (n=13) were driven by the children’s views gained from the focus group interviews and gave insight into the relevant pathogenic aspects related to the disease, treatment, and later transition to everyday life, and spoke of for example long-term treatment strategies, challenges, and the possible effects of introducing DPS into the health care process [5] (Table 1).

### Design Workshops

After the stakeholder interviews, we involved 5 children who had previously participated in the focus groups in 3 explorative and generative design workshops. Each workshop was run twice, once with the 3 boys and once with the 2 girls resulting in 6 workshops in total. Around 3-4 researchers participated in each workshop, where one child and one adult always cooperated, and the remaining researcher functioned as facilitator. Each workshop lasted 3 hours including a meal break (Table 1).

Comics were used as a theme throughout all workshops. Having a familiar theme recur throughout all workshops can serve to make the design process easier for the participants to understand [32]. Each workshop had a different focus: (1) building familiarity and creating proxy personas, (2) creating redemption scenarios, and (3) feedback and prototyping. After both rounds of workshops had been performed, the 7 children who had participated took part in a summary session where they gave feedback on the outcomes and early prototypes. This also served to inform the children of what their contribution had contributed to, as is recommended from an ethical standpoint [26].

A few months after the conclusion of the explorative design workshops, a fourth validation workshop was conducted with 7 children aged 10-12 years. These children had not been part of the previous design workshops or focus groups and did not have a history of cancer. The work in this workshop was performed in groups of 2-3 children with 1-2 adults for 3 hours. The aim of this workshop was to gain feedback related to feasibility on presented design concepts.

#### Workshop 1: Proxy Personas

The aim of the first workshop was mainly to create familiarity within the group, as not all participating researchers had met the children. The aim was to create proxy personas that the participants could identify with and that could be used as the base of the redemption scenarios of the second workshop.

Each child-adult pair created one cut-out-doll character and presented their character to the rest of the group at the end of the session. During the character creation process, the facilitator motivated the participants by asking questions about the characters that the pairs were creating. These questions ranged from concrete and tangible (eg, “what is your character’s name?”) to more value-oriented and abstract questions (eg, “what does your character like or dislike?” and “what is important to your character?”). The latter questions were informed by the focus group interviews, as well as the information gained from the stakeholder interviews. From this work emerged basic demographic information as well as values and motivational aspects of the characters. This information about and descriptions of the characters were after the workshop summarized and compiled into visually appealing and informative descriptions that we called proxy personas.

#### Workshop 2: Redemption Scenarios

The aim of the second workshop was to create redemption scenarios in the form of stories, using the characters created during the first workshop. Initially, the children were asked if they wanted to change anything about their characters. When they were satisfied with the characters, they each again teamed up with an adult and set to the task of completing comics. This process is similar to that detailed in [33]. The pairs were given the beginnings and endings of comics that detailed scenarios that were initially problematic (eg, the character feeling that their friends didn’t understand them) and eventually were solved through social interaction (eg, the character feeling that their friends understood them better). The pairs were asked to fill in the middle, that is, how the problem was solved.

Each storyline was set a priori based on theories of friendship and peer support [4] and the conceptual model of friendship established from the focus group interviews [27]. The purpose of the task was to gain the children’s perspective on how these positive experiences were created. The comics allowed the design teams to explore themes such as friendship, social support, and play. Yet, by using comics and characters as proxies, the children were not speaking about themselves and their own experiences, thus reducing the risk of their participation being upsetting.

The story-creation process allowed us to learn about the children’s reasoning and preferences for social interaction, and how they currently received support from both family and peers. The process corresponds to context scenario creation [14], where the characters act as personas.

#### Workshop 3: Design Session

The focus of the third workshop was ideation. The group with 3 boys was asked to give feedback, discuss, and change design concepts presented as continuations of the comics they created in the second workshop. The group with 2 girls was asked to sketch and prototype design solutions that fit into the comics’ redemptions scenarios. The aim was not to identify more design ideas but to keep exploring the children’s interests and motivations. Therefore, the ideas were allowed to vary in both theme and quality. Whereas some concepts showed promise, others were not realistic. Nevertheless, all ideas were captured and made part of the subsequent analysis.

#### Workshop 4: Feedback on Feasibility

The fourth workshop was, as mentioned, conducted with a group of children who did not have a history of cancer and had not previously been part of the research project. The aim of the workshop was to gather feedback on suggested design concepts. We chose to include healthy children at this stage to not overuse the limited target group [26], and the sought feedback was not unique to this target group.

The children worked in teams of 2-3 and moved between 3 stations. Each station was run by 1-2 adults and was given approximately 30 min. The concepts presented at each station were in the form of low-fi prototypes, and the children gave both verbal and drawn feedback. The feedback varied from simple adaptations to completely new design concepts, both feasible and unfeasible. The workshop was concluded with an open discussion in the entire group.

### Modeling

The modeling phase comprised qualitative analysis of the collected data [34] and is summarized in Table 1. Two kinds of models were the primary outcome from this phase: personas, and context and key-path scenarios [14]. These models guided the identification and exploration of a number of design concepts and also played an important role in the further design work; for example, by allowing us to maintain a user focus in the stages where users were not actively involved. The empirical data with which we entered into the modeling phase consisted of:

- A conceptual model of friendship from the perspective of children with experience of cancer treatment (from the focus group interviews)
- The pathogenic perspective of the children’s experiences (from stakeholder interviews with health care professionals and parents)
- Proxy personas (characters) cocreated with children (from design workshop 1)
- Redemption scenarios depicting stories about friendship, social support, and sensitive contexts, cocreated with children (from design workshop 2)
- Prototypes of design solutions that fit the redemption scenarios, cocreated with children (from design workshop 3)
- Dialogue and interaction between children, researchers, and designers, developed and deepening over 3 design workshops (from design workshop 1-3)
- Feedback on and adaptations of design concepts by children (from design workshop 4)

**Table 1:** Summary of the empirical data from the discovery phase (step 1-3) and the persona construction progression in the modeling phase (step 4).

Steps	Focus groups	Stakeholder interviews	Design workshops	Modeling
Participants	5 groups with in total 5 girls and 10 boys, 8-12 years old, and treated for cancer	13 interviews with in total 9 health care professionals and 4 parents of children treated for cancer	2 groups with in total 2 girls and 3 boys, 11-13 years old, and treated for cancer, participated in 3 sequential workshops 1 group with in total 3 girls and 4 boys, 13 years old, without experience from cancer, participated in a final workshop	
Types of data	Interview transcripts Video footage	Interview transcripts	Characters (proxy personas) Redemption scenarios (comics) Video footage	Affinity diagrams
Findings	Conceptual descriptions of friendship	Roles and effects of a DPS^a^ from a pathogenic perspective “Wicked” design challenges Onboarding and positioning of service in relation to existing health care processes	Scenario insights on behaviors, attitudes, motivations regarding friendship, relations, peer support, and redemption strategies	Categories and behavioral dimensions for boys and girls in the user group
Progression of persona construction	Discovering central salutogenic concepts (ie, friendship, peer support) that need stakeholder input	Defining the context (ie, vulnerability, friendship with peers, and surrounding environments such as hospital, home, and school Friendship, cancer or disease aspects	Building character and story: Narrative constructs based on the concept and context captured in scenarios	Complete primary personas (Anton, Julia, and Anna)

^a^DPS: digital peer support.

The data from the focus group interviews were analyzed using a qualitative content analysis approach [35]. The analysis process produced a conceptual model of friendship, with the specific perspective of children with a history of cancer [27]. This model consisted of three generic categories: (1) “common interests and experiences,” (2) “mutual empathic actions,” and (3) “mutual trust and understanding” that describe the progressive process of becoming friends. The model was central in the subsequent work, including the framing of the themes for the stakeholder interviews and the redemption scenarios during the design workshops.

The qualitative analysis of the stakeholder interviews identified a number of so-called “wicked problems” of design in this context [5]. There is, for example, the issue of screen time, where we want to encourage outdoor physical activity to improve the children’s wellbeing, yet are at risk of increasing screen time by the introduction of a new digital service. The analysis of the stakeholder interviews further identified a suitable onboarding process for the DPS service and positioned the service as strategic and salutogenic [5].

The design workshops were analyzed qualitatively in order to model the rich gathered data [36]. The proxy persona descriptions and comics were combed for details on behavior, motivation, goal fulfillment, technology use, social media interaction, social interaction, and communication channels (analog and digital). Three researchers wrote down all terms relating to this aspect that they could identify in the material, and to this the pathogenic input from the stakeholder interviews was added, along with additional concepts derived from quotes from the dialogue during the design workshops. All terms were written on post-it notes and assembled on a wall, where they were grouped by similarity in an iterative and collaborative fashion. Each change was made with an articulated intention (eg, “I’m moving term X to category Y because of Z”).

From this, clusters emerged and formed categories into an affinity diagram [36]. A total of 40 categories were formed and these were in turn organized into eight themes. For example, the theme “creativity” contained the categories building, drawing, and taking photos. Some categories in turn contain subcategories with multiple connections. The category “building” is for example separated into digital construction (with atomic data points such as Minecraft) and analog construction (eg, playing with Lego or building a tree house). Minecraft also belongs to the category computer games in the theme “consume,” whereas building a tree house also belongs to the theme “physical outdoor activities.”

The rich, multi-level affinity diagram that was created at this stage formed the basis for the creation of authentic, high-quality personas [14,36,37]. It was also noted early that certain categories were intimately tied to gender; several apparent differences between the data from the two groups of children emerged. For example, from both groups, the themes of technology, creativity, and social interaction were identified, but the girls were more oriented toward photography and sharing photographs on Instagram, whereas the boys built Minecraft worlds while communicating using Skype.

## Results

### Coconstructed Child Personas

**Figure 1:**
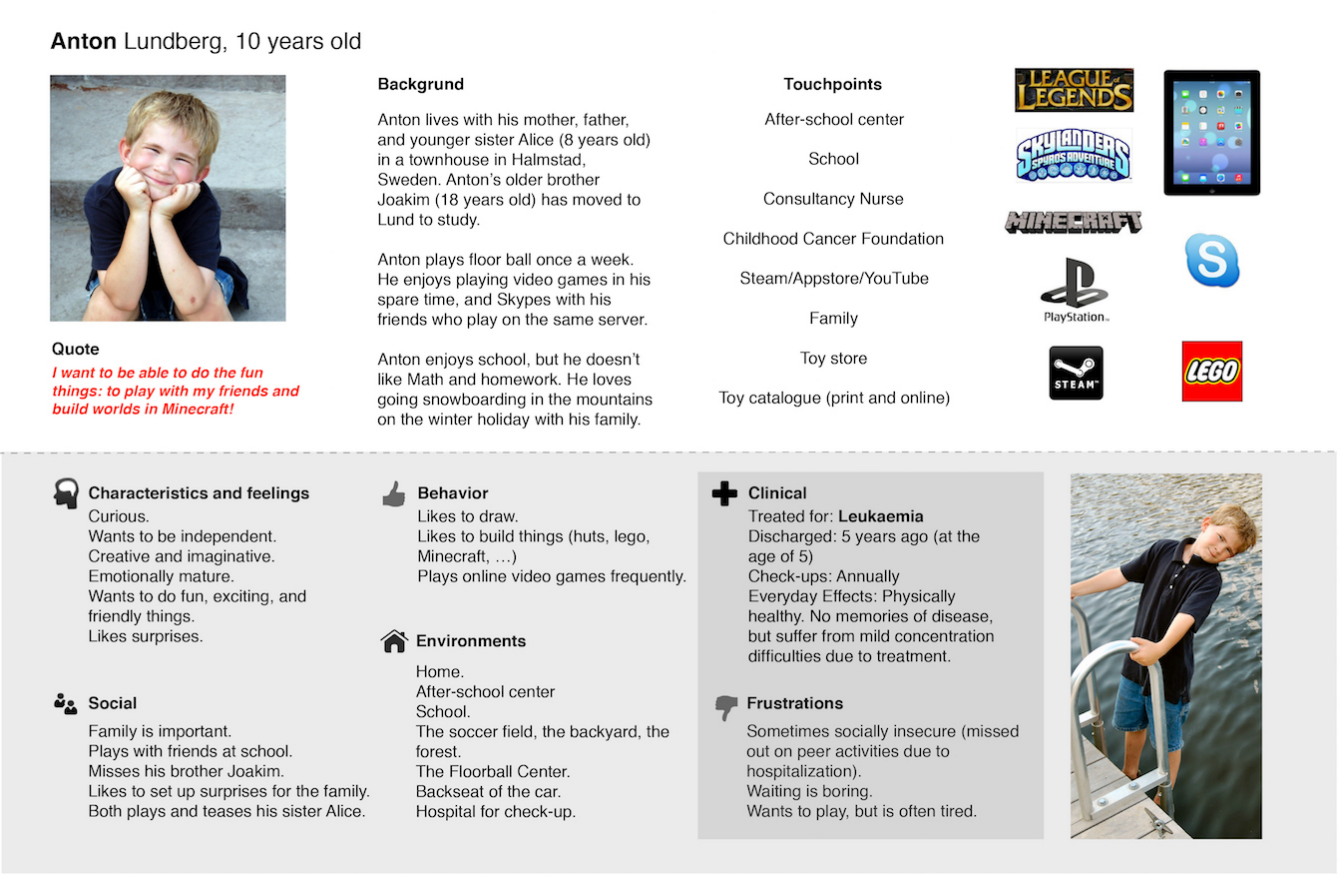
Persona description (translated from Swedish) of Anton, one of the project's three primary personas. The persona description merges the pathogenic perspectives (summarized in "Clinical" and "Frustration" categories) with the salutogenic perspectives.

Following the persona-creation process advocated by Wärnestål et al [34] and Cooper [14], three primary personas were created: 1 boy (Anton) and 2 girls (Julia and Anna). The main distinction between them was their main goal for interacting with the DPS service. Table 2 shows a summary of the project’s three primary personas, covering the complete service lifecycle from initial user onboarding (Anton), through continuous use (Julia), to an “alumni” perspective after exiting the peer support service (Anna). Figure 1 shows the overview page of Anton’s persona description.

The progression of the personas’ richness is based on the four steps summarized in Table 1. The persona descriptions of Anton, Julia, and Anna contain interpreted data from focus group interviews, stakeholder insights derived from the interview analysis, as well as a distillation of the proxy persona attributes and social redemption scenarios discovered in the 3 design workshops. Of particular interest for child-persona construction in this design context are results relating to: (1) pathogenic versus salutogenic perspectives, (2) platforms for Web-based communication, and (3) implications for avatars and conversational user-system interaction.

**Table 2:** Persona overview of the three primary personas.

Persona A	Persona B	Persona C
“Anton” Male, 10 years old	“Julia” Female, 12 years old	“Anna” Female, 16 years old
Role: Player (entering the DPS^a^ service)	Role: Mentor (experienced player)	Role: Alumni (leaving the DPS service)

^a^DPS: digital peer support.

### Salutogenic and Pathogenic Perspectives

The personas manifest the synthesis of the pathogenic and salutogenic perspectives. The pathogenic orientation is most evident under the “clinical” and “frustration” headings of the persona descriptions. The other four headings are mainly derived from the salutogenic orientation. However, content in these headings are verified and complemented by pathogenic perspectives. For example, the characteristic “emotionally mature” was verified by both parents and health care professionals. The characteristic “wants to play” (described by children, and part of Anton’s persona) was complemented with “but is often tired” in the “frustrations” heading from parent interviews. The result of such syntheses emphasizes the necessity of both the salutogenic and pathogenic perspectives when developing personas for a DPS service in this domain.

### Child-Centric Views on Web-Based Communication

**Figure 2:**
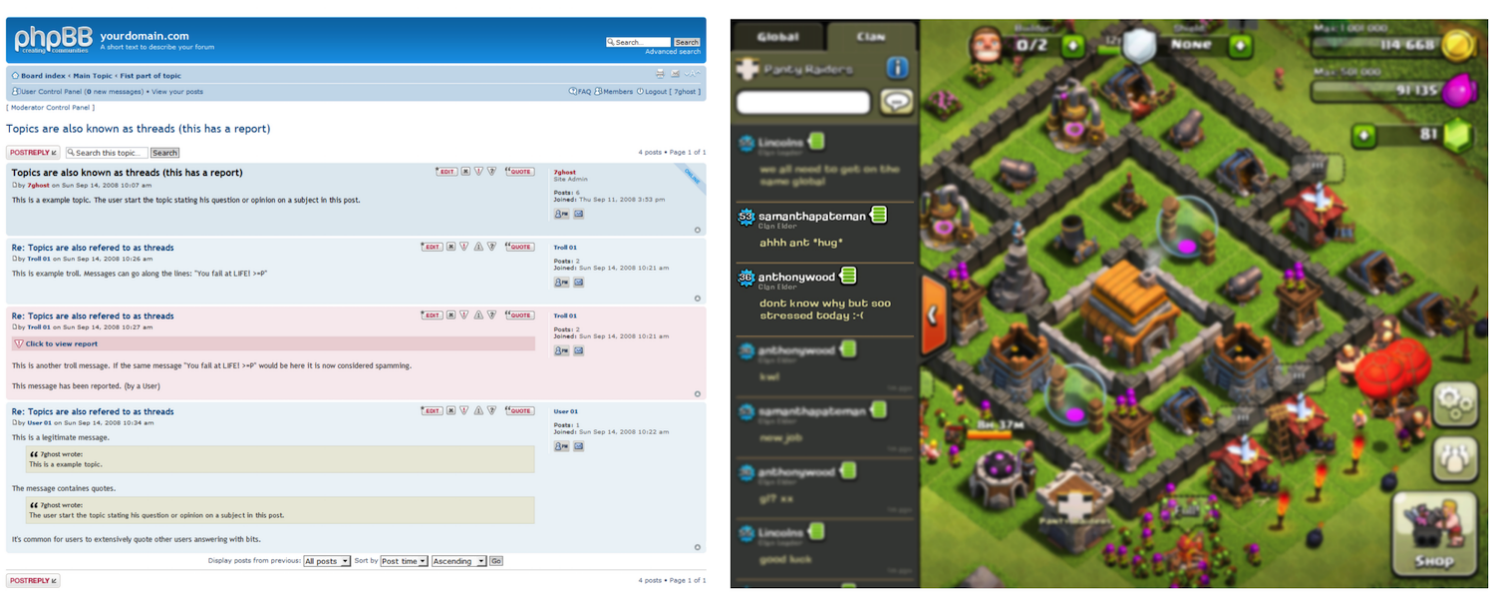
Comparison between text-based forum (generic legacy phpBB on desktop web browser, referenced in the stakeholder interviews), and chat in the tablet-based game Clash of Clans (referenced in the children workshop sessions). Example of supportive chat between the players is highlighted in the game interface (e.g. second and third utterances in the chat area to the left in the Clash of Clans interface).

Due to its qualitative and rich nature, a number of child-centric insights that ran counter to what we learned in the interviews with adults (parents and health care providers), surfaced in the workshops. These aspects were incorporated in the personas to maintain an authentic end-user voice in the service design process that followed the persona generation. Figure 2 shows one such difference between child and adult perspectives on Web-based communication.

In the interviews with adults, the topic of “Web-based communication” between children came up several times. However, the references and examples used ranged from text-based forums (such as phpBB in Figure 2), mature social media platforms such as Facebook, to even more traditional media such as telephone calls. This stands in contrast to the media choices introduced and referred to by the children. Facebook was not used by a single child in our study, and the preferred communication platforms were either in-game conversations (such as the Clash of Clans chat interface in Figure 2) or newer social media platforms such as Kik and Snapchat. The two latter platforms were mentioned at all by neither health care providers nor parents.

### Design Implications for “Give Me a Break”

**Figure 3:**
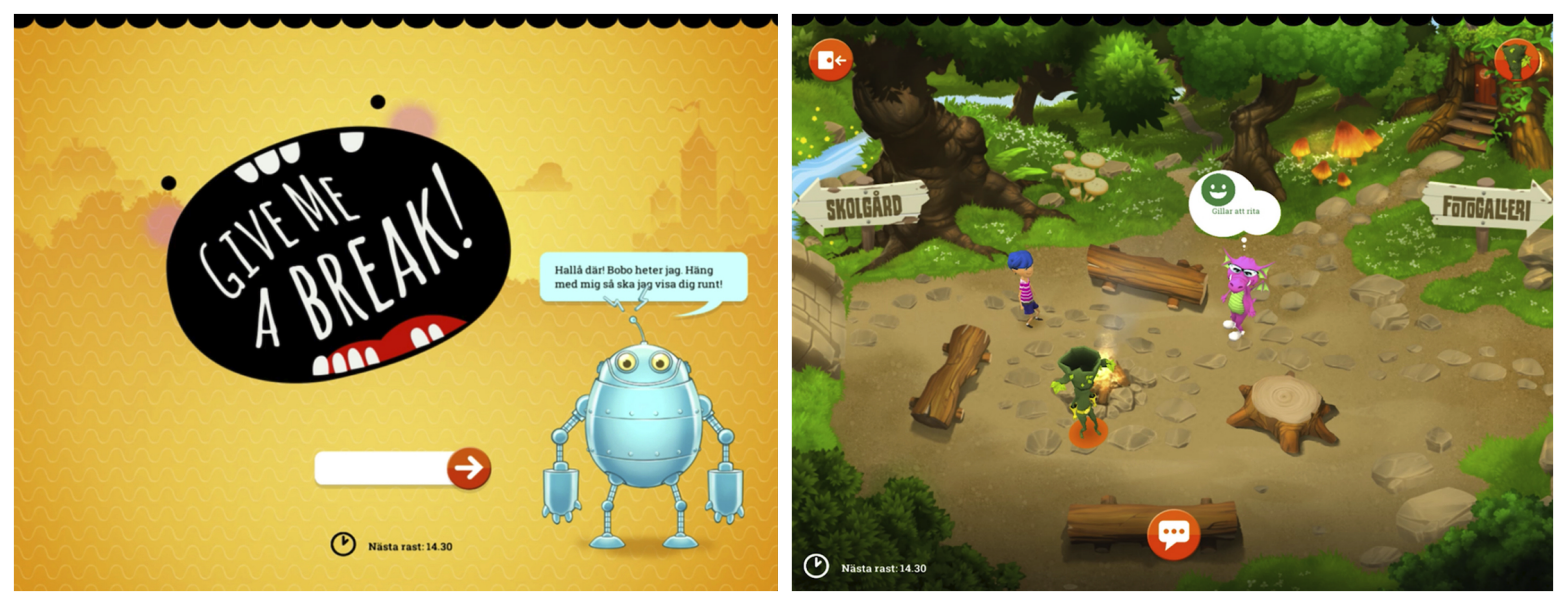
Screenshots (a, left) and (b, right) from a Swedish high-fidelity prototype of the digital peer support (DPS) tablet app “Give Me a Break.” Screenshot (a) shows the user’s first encounter with the tutor robot “Bobo” in the onboarding process. Screenshot (b) shows the virtual environment where player avatars display their interests in thought bubbles in order to spark interest and conversation. In this particular screen, the purple dinosaur displays the user’s interest in drawing.

Give Me a Break is a tablet-based, Web-based service developed for childhood cancer survivors based on the three personas described in the previous sections of this paper. The service is a functional prototype and has been tested with real users [38]. A detailed description of the service is beyond the scope of this paper. However, we highlight some features of the service that exemplify the effects of using the coconstructed personas. Figure 3 shows the gradual revelation of the onboarding sequence. Bobo, a friendly and conversational robot, greets the user and explains the sign-up process in discrete steps. This solution is based on the Anton persona’s need to carefully understand things in manageable steps, and all three personas' desire to engage in conversation rather than standard form-filling sign-ups. Bobo helps the user craft a Web-based avatar that the user navigates the virtual environment with. Bobo follows the user into the game world and explains the core concepts that the user can explore. As the user gets acquainted with the mechanics of the game experience, Bobo’s presence diminishes to let the user focus on conversational interactions with others instead. Figure 3 shows one of the scenes in the environment. In order to trigger conversations between users, which are the underlying mechanic to achieve peer support effects through friendship [26], user avatars display content from their respective interests listed in their profiles that Bobo helped set up in the onboarding process. By automatically displaying a user’s interest thought bubble (eg, “I like to draw” or “I love to play Minecraft”), the avatar provides an actionable conversation starter for surrounding users. This resonates with both Anton and Julia, who both are driven by social interaction, but are cautious around new people and sometimes unsure of how to approach others.

## Discussion

### Principal Findings

The methodological implications for a child-centric design process in this study is that a UCD process needs to be customized to fit the design space for (1) child users; (2) the domain of health-promoting and digital, social services; and (3) sensitive contexts. These three qualities are not explicitly addressed by generic persona-driven methods developed for adult, professional users in a productivity- or efficiency-oriented domain. The traditional principles rather regard goals such as minimizing work [14] and to increase productivity and reduce costs. For children’s interaction with technology in terms of digital toys, games, and other more experience-oriented products, such as social media platforms, principles beyond productivity and effectiveness have to be acknowledged [39,40].

Cocreative aspects have been voiced as an important part of child-computer interaction, such as Druin’s work on cooperative inquiry [7,41], and the initiatives stemming from the Scandinavian participatory design approach [9,42]. Such advances address the needs of developmental abilities of children when involving them in the design process. However, it is difficult to directly implement standard interview and participatory design techniques for young children because of difficulties with abstraction and verbalizing conceptual problem solving [39]. Furthermore, in the context of designing for children in sensitive contexts relating to disease, there are pathogenic aspects that designers and researchers need to incorporate in any design efforts for the target group, that need to be collected in other ways due to ethical reasons as outlined previously. In our approach, the synthesis of salutogenic and pathogenic input to the child personas help designers to maintain an authentic user-centered approach in such contexts.

The rest of this section discusses the effects of using the personas in the design of a DPS service for childhood cancer survivors. First, we discuss how personas were used as communication aid within the design team, as well as for outside communication of the project. Second, experiences on how personas worked as an idea-generating tool are described. Third, we describe how personas were used as tools for making design decisions throughout the design process.

### Personas as Communication Aid

Due to their familiar narrative-based form, persona descriptions provide a common language for discussing effects of the design. By referring to the primary personas, a consistent picture of the target user group could be formed. Personas were used as communication aid between a wide range of disciplines and roles (interaction design, research team, care-givers, parents, game developers, and so on), and helped build a common vision in the within an interdisciplinary team and external actors.

#### Within-Team Communication

Within a multi-disciplinary group, comprising researchers and practitioners from nursing, medical science, and human-computer interaction—as well as children users—character and story are the “lowest common denominator.” Personas and scenarios provide the lingua franca for service innovation and development, where designers, researchers, children, parents, and caregivers have a common ground in the conversation about the service. Since the children had taken a very active part in the development of the proxy personas and scenario ideas, it also empowered them in the design discussions.

The persona descriptions also help enforce a “project language,” which is necessary to avoid misunderstandings and misinterpretations. We found that using personas and accompanying scenarios formed a productive foundation for workshops and idea generation sessions. Furthermore, the use of personas helped us communicate around the design’s effectiveness.

#### Involving External Actors

External collaborators (such as the game production company that built the first high-fidelity prototype of the DPS service, or business investors) clearly understand the essence and intent of the service when presented with personas and scenarios.

Personas and scenarios were used effectively in business modeling workshops for communicating and developing business models surrounding the service. By employing a narrative in the form of scenarios where the primary personas interact with the service, it was clear how business model decisions could affect the user experience of the service. This allowed us to measure the design’s effectiveness and see the implications in terms of organizational structure and other strategic planning activities related to the DPS service development.

The embodiment of the users’ goals and needs makes it easier for the team to focus on the common understanding of the end users. This is especially important in multidisciplinary teams, as well as when interacting with external actors and developers. The personas therefore contributed to building consensus and commitment to the design.

#### Personas as Catalysts for Dealing With Legal and Ethical Positioning

Our three personas functioned as rich archetypes, which effectively steered the design team members away from stereotypical renderings of the user group. The persona descriptions helped both stakeholders and designers to reduce the users to caricatures, and instead allowed the team to talk about the rich and authentic personalities of Anton, Julia, and Anna. By relating to and designing for authentic people (fictional characters—yet based on rich empirical data), both researchers and designers voiced that they increasingly understood and cared about what would happen to the personas given a specific design suggestion. In short, the team developed empathy for the personas, in a way that the elastic concept of “the user” would not. This was evident in the discussions regarding ethical and legal positioning. By thinking and reasoning about the consequences of a design decision in terms of an authentic user, new aspects were uncovered that could be addressed at an early stage. One clear example of this is the way that the members of the team routinely would ask: “How would Anton feel if the service was implemented this (or that) way?”

### Personas and Scenarios as Idea Generators

Having personas based on salutogenic input from the children, it was natural to evolve the solution into playful interaction based on positive health outcomes. This complemented a more traditional approach of letting stakeholder input (which in this project would have been mostly pathogenic) dictate design decisions. Instead, the pathogenic aspects served as a backdrop to check ideas generated from the primary salutogenic persona needs.

Positioning, wicked problem identification, and onboarding (stakeholder input) is not enough to provide a solution that resonate with this target group. The salutogenic perspective, the children’s point of view regarding what kind of service they want to have, and the interactive and aesthetic qualities (ie, what the children are used to and what they appreciate in terms of digital service interaction with games and other apps) were not captured in a relevant form in the stakeholder interviews. It proved to be quite the opposite: both parents’ and medical professionals’ views on digital interaction and digital services were sometimes both naive and outdated. Phrases such as “what kids do on the Internet these days is beyond me” and “I don’t understand all these new apps that the kids are using” are examples of this sentiment. In the interviews, our stakeholders referred to text-based discussion forums and Wikis as possible platform ideas for the service, whereas the social interaction platforms that were suggested by the children codesigners never even mentioned these. Instead, they mentioned more contemporary social media platforms, as well as in-game chats. Figure 2 illustrates the difference in reference frames between the stakeholder and end-user (children) input.

Scenarios that exemplify how Anton, Julia, and Anna interact with relevant social media services (as of early 2016 these include Snapchat, Instagram, Skype, Facetime, and various in-game messaging solutions), could then serve as a means of communicating this effectively to the stakeholders.

### Persona-Driven Design Decisions

The developed personas guided design decisions of both major significance for the whole project and at a level dealing with minor design decisions regarding function and aesthetics of the digital service. In this section we exemplify how personas influenced the service design at these different levels. Example 1 is a major service design decision, helping setting the entire vision of the project. Example 2 is a medium-level service design decision that highlights how the stakeholder interview analysis gives insight on when and how the onboarding takes place, modified by persona behaviors and characteristics, rendering the onboarding scenario tailored to fit Anton’s profile creation. Example 3 is lower-level interaction design decision, helping enhancing usability and user experience in the initial interactions in the platform.

#### Example 1: Shaping the Entire Service Experience

An important synthesized insight from children dialogues and stakeholder input is the need for training social skills for this particular user group. Since they have missed large portions of time in school due to being hospitalized, they miss opportunities of training social skills during school breaks and extracurricular activities. In the words of the children themselves: “on the breaks you learn how to have fun with friends.” This, along with interviews with young adults that want to reconnect with lost acquaintances on other platforms, the idea of a (long-term) virtual real-time playground emerged. By examining the different attitudes, interests, and behaviors of the three primary personas, it was clear that newcomers and “old-timers” would have different needs and skillsets on a DPS platform. Hence, the idea of player, mentors, and alumni emerged and were integrated into the design of Give Me a Break. These roles were crystallized and refined as a direct result of scenario building around the personas and shaped the entire peer support service to be centered around player onboarding, mentor interaction between both players and mentors, as well as supporting contact facilitation between alumni in other channels and platforms after they have stopped using the original peer support service.

#### Example 2: Addressing Persona Behaviors in the Onboarding Context

During service onboarding, the initial concept was a fairly standard user profile setup. Setting up user profile interests was initially designed as a “wizard-style” step-by-step interaction before entering the service and engaging with other users. When checking this procedure with the Anton persona, the team realized this was a boring, noncreative way of doing it, and stood in contrast the Anton’s behavior pattern of creating and exploring. So instead, the design team integrated the profile building as part of the playground interaction. Setting up user profile interests can now be done through a number of explorative and engaging ways, for example walking up to other people and watch their interest appear in bubbles above their avatar, or walking past an “Instagram signpost” that reminds the user to add his or her Instagram user name, or getting tips from a nonplayer helper character in the shape of a tutor robot called “Bobo” that accompanies Anton’s avatar as he is familiarizing himself with the playground environment (see Figure 3).

#### Example 3: Understandability and Ease-of-Use

For any digital service, a user needs to be able to understand the interaction design. Anton’s need to understand what to do on the playground is thus a basic usability requirement, and the challenge for a service and interaction designer is to accommodate the specific needs and provide the right kind of assistance and pedagogic vectors for Anton so that he becomes successful in using the service.

In order to design a customized experience for this particular user group, the personas were critical. Anton’s onboarding scenario, which includes offline usage mode in case Anton doesn’t have Internet connection, and the interaction design with the robot Bobo is a direct effect of (1) Anton’s onboarding context, (2) Anton’s mild insecurity due to his condition (stakeholder input), and (3) Anton’s explorative and creative mindset (salutogenic input).

### Strength and Limitations

The point of user personas is to be a useful abstraction and visualization of salient aspects of what users want, their relationship with the service being envisioned and built, and how they behave in relation to the service and other human beings. Interviews and workshop material provided us with patterns and insights regarding attitudes, goals, behaviors, skills, and needs of our users. Our three personas represent behavior patterns specific to salutogenic play (from a long-term, strategic perspective). As the service platform is iteratively refined and its development continuously guided by user tests, new insights can naturally be incorporated in the framework provided by our personas and scenarios.

Critique could be voiced regarding the lack of statistical significance in our findings. This critique can be met, as the kind of understanding provided by this method may not surface through a strictly quantitative approach. We argue that the method of using personas works as a complement for gaining critical insights for service design where a human interactive and social component is key. Qualitative studies put trustworthiness in focus and deals with the traditional lenses of objectivity, dependability, credibility, relevance, and transferability [39,43].

### Conclusions

The motivation behind the research reported on herein stems from methodological constraints put forward by the specifics of vulnerable children in health-related, sensitive contexts. As design thinking and UCD practice find their way into processes that aim to cater for patient experience in health care [44], there is a growing need to customize methods and techniques originally devised for more traditional applications and domains.

This paper provides insights and resolutions to difficulties that can arise when empirical data from end users are restricted due to clinical and ethical reasons. By combining the salutogenic (from the children) and pathogenic (from adult stakeholders) perspectives, we could learn about aspects related to both health and disease that are important for the design of authentic, child-centered personas that could be employed for investigating complex themes of friendship and wellbeing, peer support and relationships, and the role of social technology in daily life. The design-oriented method described in this paper is ultimately about understanding human behavior in relation to a specific situation and context. It is therefore applicable even in processes where a digital artifact or service is not necessarily the outcome.

## Abbreviations

DPS: digital peer support
GDD: goal-directed design
UCD: user-centered design
